# No-boundary thinking: a viable solution to ethical data-driven AI in precision medicine

**DOI:** 10.1007/s43681-021-00118-4

**Published:** 2021-11-29

**Authors:** Tayo Obafemi-Ajayi, Andy Perkins, Bindu Nanduri, Donald C. Wunsch II, James A. Foster, Joan Peckham

**Affiliations:** 1 Engineering Program, Missouri State University, Springfield, MO USA; 2 Department of Computer Science and Engineering, Mississippi State University, Starkville, MS USA; 3 Department of Comparative Biomedical Sciences, College of Veterinary Medicine, Mississippi State University, Starkville, MS USA; 4 Electrical & Computer Engineering Department, Missouri University of Science and Technology, Rolla, MO USA; 5 Biological Sciences Department, University of Idaho, Moscow, ID USA; 6 Computer Science & Statistics Department, University of Rhode Island, Kingston, RI USA

**Keywords:** Data science, Precision medicine, Ethics, Artificial intelligence

## Abstract

Today Artificial Intelligence (AI) supports difficult decisions about policy, health, and our personal lives. The AI algorithms we develop and deploy to make sense of information, are informed by data, and based on models that capture and use pertinent details of the population or phenomenon being analyzed. For any application area, more importantly in precision medicine which directly impacts human lives, the data upon which algorithms are run must be procured, cleaned, and organized well to assure reliable and interpretable results, and to assure that they do not perpetrate or amplify human prejudices. This must be done without violating basic assumptions of the algorithms in use. Algorithmic results need to be clearly communicated to stakeholders and domain experts to enable sound conclusions. Our position is that AI holds great promise for supporting precision medicine, but we need to move forward with great care, with consideration for possible ethical implications. We make the case that a no-boundary or convergent approach is essential to support sound and ethical decisions. No-boundary thinking supports problem definition and solving with teams of experts possessing diverse perspectives. When dealing with AI and the data needed to use AI, there is a spectrum of activities that needs the attention of a no-boundary team. This is necessary if we are to draw viable conclusions and develop actions and policies based on the AI, the data, and the scientific foundations of the domain in question.

## Introduction

AI has a long and rich history as a sub-discipline of Computer Science to automate intelligent computational approaches that are as close to human problem solving capabilities as possible. However, the term AI has recently been conflated with machine learning, and especially deep learning. Machine learning is a computational approach in which algorithms are developed to learn from data. The algorithms identify patterns or rules to find trends or signals in data in order to make predictions or perform analysis of future data, or to explain the data. Rules guiding analysis are not given to the algorithm by the human programmer. The programmer codes the algorithm that infers the rules, thus the algorithm learns from the data and uses this for future analysis and predictions [1]. Deep learning is a sub-field of machine learning that uses multiple layers of artificial neural networks, a specific machine learning technique that is based on an “artificial” and simplified model of how living animal brains might work. To avoid confusion, we adopt the currently popular meaning of AI as machine and deep learning, although our argument posed here is broadly applicable to computational tools that rely upon data.

Today AI is used to help us make difficult decisions about business, policy, health, and our personal lives. We have learned that both data and algorithms can be imbued with the same prejudices and oversights that humans harbor. Policies that encode and perpetrate racism, for example, can be harmful [2]. Bioinformatics and health informatics are closely related and overlapping interdisciplinary domains for which there are serious ethical considerations. Health ethics has mostly focused on the just application of health practices to preserve the patient’s autonomy, safety, and privacy. However the rise of intelligent automated tools to guide health practitioners brings another dimension that begs for attention. For example, in light of the COVID-19 pandemic [3], a key ethical question is how to integrate machine-supported statistical analyses and human judgement to weigh loss of lives and livelihood to choose the best course forward.

Current challenges of AI in precision medicine, including explainability [4] and interpretability of AI techniques, emphasize the need for this paper, given the ethical implications of misunderstanding results [5]. Precision medicine is “an emerging approach for disease treatment and prevention that takes into account individual variability in genes, environment and lifestyle for each person” [6]. This necessitates advanced approaches to collect, manage and analyze large and diverse data sets about individuals, including biological, genetic, environmental, and behavioral data, and information about prior conditions, and responses to treatments. If we do not get these things right, we risk misinterpretation of data upon which diagnoses, treatments, and policy decisions are made. This could have serious ethical consequences for patients and providers. Ethical problems are usually unintended consequences of the application of the technology [7]. The usual ethical approach is consequentialist (see Sect. 2.2) in nature, which implies that we use and interpret AI tools to inform decisions that support the well being, dignity and rights of groups and individuals, and aim to do the least harm. The question then arises, how do you determine the “least” amount of harm or is no harm possible [4]?

We make the case that a no-boundary thinking (NBT) approach [8]^1^is essential. NBT is a novel method for scientific discovery and education that accesses and synthesizes knowledge from all disciplines to define important problems [9, 10]. This leads to innovative and impactful questions that can be addressed by individuals or teams with diverse expertise. The no-boundary approach helps to remove the emphasis from data analysis and places it on problem definition, involving a variety of stakeholders. In machine learning approaches, NBT is vital to all steps, including feature selection, training, and interpretation and translation of results. Domain knowledge is essential to each of these steps, but failure to consider socio-cultural, legal, and ethical aspects, for example, can lead to results that might not be optimal. These results may be at best short-sighted and at worst biased or discriminatory. The NBT approach argues that expert attention to algorithms and data are equally important, as is the interaction between AI experts and domain experts.

Attention to possible flaws in the conduct and application of AI is important and the development of no-boundary or convergent approaches is essential. While experts have rightfully focused on fairness in algorithms [11–13], there is a whole range of data related activities [4] in support of algorithmic analysis that needs similar attention. Our overall objective is to help stakeholders to address the ethical implications of data science and AI in medicine by employing NBT approaches. This paper examines the use of AI to carry out automated and intelligent data analysis of healthcare data for advancement of precision medicine. We identify potential ethical risks, explore different ethical theories, and urge consideration of approaches to assure that we are “doing the right thing” when applying AI to precision medicine. Our position is that AI has great promise in precision medicine, but we need to move forward with great care, with consideration for the possible ethical implications, and develop AI approaches and processes that support ethical AI. The remainder of the paper is organized as follows. We provide a short review of the foundations of data, algorithms, and ethics in Sect. 2. Section 3 explores how convergent or no-boundary thinking is essential in supporting the ethical use of AI in all intelligent systems, particularly in the domain of precision medicine. We discuss open problems and questions in Sect. 4 and conclude in Sect. 5.

## Background

The COVID-19 pandemic provides many examples of the ethical quandaries that may arise when we use data and algorithms to make critical decisions. How do we integrate machine analyses and human judgement to weigh loss of lives against specific approaches to slow the spread of the virus? The multiple shutdowns experienced globally shed light on the difficulty of determining the balance of shutting down industry and trade against loss of lives to the virus. How do we use intelligent analysis to weigh loss of livelihood against loss of life? Can we or should we try to encode this into a fair algorithm that supports ethical decisions? [3] How do we collect, clean, organize, and access the data that provides informative input to these “intelligent” processes? And what assurances do we have that these algorithms are fair? Do we know the likelihood of error, and can we uncover biases in the data and algorithms? What is our responsibility for communicating to stakeholders and decision makers what the AI and data are telling us? Especially importnat in precision medicine, how do we assure that all of this is ethical, does no harm to individuals, companies, government, families, and society? Each approach can save lives and keep individuals happy, healthy and safe, but each brings consequences that may negatively affect the well being of society, industry, and individuals. It seems clear that answering these questions will require experts from a diverse range of disciplines, a no-boundary approach.

There are many places in the application of AI where things can go wrong. For example, [5, 12, 13] and [3] have documented such places in the collection, handling, and interpretation of data that could be problematic. In this section, we examine some of these data, algorithmic and domain considerations.

### Role of data

Our push to advance understanding of clinical data has driven the development of new and increasingly sophisticated analysis tools. According to Mesko, “There is no precision medicine without AI” [14]. Advances have been made in predicting risk for some diseases using multidimensional data, but challenges remain, and more development is needed for a broader range of other disorders [15]. Digital technologies are increasingly used in self-care, clinical care, and biomedical research, and it is important for developers to consider ethical components in the design process [16].

An AI is an intelligent algorithm powered by data and enabled by robust data models and AI models. We model reality to make sense of biomedical information, and rely on data and models as a basis for important medical decisions. We cannot capture every detail of that which is being modeled, thus every model is flawed, so interdisciplinary perspectives are needed to identify the elements that we believe will best guide our decisions. Experts possessing this expertise are needed on the precision medicine team. This includes data modeling expertise to guide the organization of data for archival, access, pre-processing, and correct and efficient access by human and machine; AI modeling expertise to choose appropriate algorithms and frameworks for deriving predications and results; and biomedical domain expertise.

Diverse expertise is also needed to develop techniques to identify characteristics of data sets that work best with a particular analysis model, calculate the probability of error, and increase the probability that we have accurate and meaningful results. While statisticians have developed well formulated constraints that guide which data sets are appropriate for a given analysis technique and methods for estimating likelihood of error, computational models do not yet have precise mechanisms for such estimates, and rely more on human expertise and judgement to match the algorithms and their constraints to the characteristics of the data. Further, the analysis approaches taken by algorithms are frequently buried in code and less transparent than the mathematical equations of statistics.

Statistical and computational approaches are facing greater challenges in this era of Big Data [17] that presents messier, larger and increasingly complex data sets, compelling experts to develop intelligent computational approaches for data sets that do not yield to traditional approaches. AI analysis of biological and medical data [14, 18–21] brings with it a special set of difficulties that parallel many of the oft cited challenges of Big Data as follows. These include issues of voluminous data, dirty data as a result of the biological collection process, sparse data due to insufficient samples, as well as computationally complex analyses, such as in pharmaceutical drug discovery environments where models are highly complex.

This paper urges NBT attention to data in its broadest context including collection, cleaning, curation, sampling, and archival, as well as the application of analysis algorithms to data to make discoveries, build predictive models, interpret, and communicate results. In the domain of precision medicine, if there is a flaw in any of the steps of data procurement and organization, or a mismatch among data, analysis model, and algorithm, we are left with potentially dangerous or life threatening consequences. In the next section we consider ethical foundations that could inform how we proceed with AI in precision medicine.

### Ethical foundations

In order to enable a more objective and thorough consideration of the ethical issues posed by modern technologies, it helps to understand the foundations of ethical theory, a subject that has been studied for millennia in Philosophy. It is also useful to distinguish between the ethics of AI and AI ethics, since this distinction is rarely made outside the philosophical literature [22]. The distinction lies in who the ethically relevant agents are. In the ethics of AI, we examine the ethical constraints on bioinformaticians and engineers, those of us who do AI. AI ethics, on the other hand, examines the ethical constraints on the artificially intelligent artifacts themselves. In this paper we examine the ethics of AI. But the survey below is relevant in both contexts. (This section is a limited review; see [23] for a more detailed overview.)

A narrowly consequentialist view reduces ethical analysis to a discussion about what counts as harm or benefit, how to measure it, and whose interests matter. Consequentialist ethical theories presume that the consequences of actions determine their ethical status. Consequentialist schools differ according to what consequences matter. Utilitarianism argues that ethical action maximizes benefits such as happiness or minimizes harms such as suffering. Consequentialist theories analyze what should be optimized and how. This is not the only possible basis for ethics. Deontological theories presume that what matters is whether acts harmonize with duties and obligations. A modern shadow is legalism, which measures the ethical status of action by how it relates to legal obligations or prohibitions, either as codified in statue or according to “natural law”.

Virtue ethics give primacy to the character of the actor. To determine the ethical status of an action, one examines what sort of person acts that way, not the action’s consequences or associated obligations. One way to pursue virtue ethics would be to reflect on what makes exemplary humans exemplary, and to emulate them. For example, most practitioners would agree that it is the responsibility of teams working with this personal genomic data to examine the implications of its use [24]. There are other ethical theories (e.g. nihilism, care ethics, situational ethics and intuitionism,) that are less relevant to this paper. It is probably not the case that only one ethical theory is relevant in every circumstance. Bioethics may tend to be deontic, engineering ethics may tend to be consequentialist, and academic ethics may tend toward virtue ethics. Computer scientists who develop artificial intelligence in bioinformatics software for clinical purpose may use some combination of all three.

When considering ethics in AI and bioinformatics, it is helpful to place any argument in the context of the ethical theory it presumes, and then to consider what sort of argument might flow from different ethical theories. Such an exercise shows respect for, or at least awareness of, ethical scholarship. It may not lead to definitive conclusions. But it is certain to improve our understanding of the issues at hand. It is reasonable to suppose that AI ethics and the ethics of AI may have different theoretical foundations. For example, it may be the case that AI robots have an obligation to not harm humans, out of respect for human autonomy. This is a deontological foundation for AI ethics. But it may be the case that AI researchers should avoid using artificial intelligence for military purposes, because the potential for harm is too great. That would be a consequentialist argument in the realm of the ethics of AI. Note that knowing the foundational outlines of philosophical ethics makes it easier to state, respect, and reason about such distinctions. In the debate surrounding AI, there is a case for the centrality of humans and their values in the development and future of AI. Some regard this as traces of humanism in that defense of human rights and human dignity could be a basis of an ethics of AI [1]. However, posthumanists question the centrality of the human in modern ontologies and ethics. From their perspective, nonhumans matter too, and we should not be afraid of crossing borders between humans and nonhumans.

## AI challenges in precision medicine: no boundary solutions

Digital technologies are being increasingly used in self-care, clinical care, and biomedical research. The role and dependence of AI (machine learning) algorithms on data for use in precision medicine cannot be overstated. In machine learning, the computer creates its own models that fit the data. The starting point is the data, not the theories. In this sense, data is no longer “passive” but “active”: it is “the data itself that defines what to do next” [1].

Machine learning algorithms can be inherently biased as a result of inadequate data, asking bad questions, a lack of robustness to dataset shift, dataset shift due to evolving health care practice, model blind spots, and human errors in design [16]. Metrics and tests that measure degree of bias of a given AI algorithm are needed. Consideration of the tradeoffs among model biases is also important, as is consideration of the ethical frameworks that guide the chosen metrics and tests.

There are many levels on which we can analyze these issues. Here we discuss in the context of a convergence of multiple disciplines for two reasons: (1) precision medicine and AI today are both areas of inquiry in which we are compelled to solicit multiple expert perspectives, so are strengthened by no-boundary approaches and (2) AI, like Statistics and computing are disciplines in which scholars develop techniques that they hope will eventually become discipline agnostic, that is part of the tool kit that scientists deploy across multiple disciplines. However, in order to achieve this, we need to understand the domains that have the sorts of problems that are in need of AI techniques. Subsequently, we outline the steps and elements needed for the creation of a robust AI for precision medicine, consider each place where ethical concerns may emerge and show why no-boundary approaches are important.

### Problem formulation and modeling

During the problem formulation and design phase, who has a seat at the table is crucial [25]. We propose a no-boundary team composed of scholars and experts from the problem domain and AI scholars and experts to consider quasi experimental or AI approaches. The initial step necessitates that the team ensures that they are asking the right question. Have they defined the ”right” problem? Then the team must develop a model of the domain that includes the variables that are considered to be important. All models represent a subset of reality so important questions arise. Have we chosen the right subset that will be informative and as accurate as needed to support our decisions? If we leave out important variables needed to capture the situation and provide important predictors, we might be making decisions that harm individuals. An example is failure to consider the characteristics of certain under-served populations when deciding appropriate medical treatments.

For example, early in the COVID-19 pandemic, in the state of Rhode Island, it took special effort to confirm through testing that under-served populations were not doing as well as others. These populations were underrepresented in the COVID-19 testing data set because initially there were few testing stations in locations that were easily accessible by these populations. This was quickly corrected, but this example highlights the critical need for AI models designed for medical applications to consider socioeconomic status as an independent variable; and include good data sampling and collection strategies.

The ultimate goal of AI in precision medicine is to arrive at clinical decisions within the current framework of physician-patient interaction. As such, issues related to patient consent, awareness and risk-benefit analysis should be given due consideration. Moreover, for implementation in the clinic, AI algorithms must be trustworthy. Accountability, transparency and governance of AI-based solutions for supporting clinical decisions are important aspects for ethical and fair adoption of AI in precision medicine. It is clear that fulfilling the promise of AI for precision medicine requires input from different stakeholders, and a convergent approach such as NBT allows evaluation of the needs and inherent biases of stakeholders interested in the outcome and holistic consideration of trade-offs.

### Data collection, cleaning, organization and access

While algorithms lie at the center of an AI, every AI needs sound data, and good processes and procedures for sampling, collecting, cleaning, and organizing data. This is important to assure that the AI is given a reliable picture of that which we are trying to capture and analyze. Each of the data wrangling steps from collection to access, have the potential for introducing errors that may influence the AI analysis outcomes. If utilizing a consequentialist ethics approach, then we must consider the consequences of each of these steps. When working with data, reliable procedures must be used. This requires a no-boundary or convergent team of expert data modelers, technicians, and machine learning or statistics experts. For example, if the data is accessed from a relational database that is not normalized, the access procedures are at risk of producing incorrectly linked or repetitive data which would influence the AI results. Under certain circumstances, a strong data scientist maybe be well versed in data modeling, analysis, and communication but lack the domain knowledge necessary to interpret and propose actions based on the on the results of the data analysis. This requires a team of individuals, each member with deep knowledge in one aspect of the problem, but also good communication and collaboration skills to support multiple appropriate perspectives on the problem.

No-boundary and convergence approaches compel teams to work together from the inception of the problem to the end, with “all hands on deck” when defining the problem. Poor problem definition and poor data handling can spell disaster for a project. For example, genetics contributes to the complexity and individual response to specific treatments. Among the various causes that result in lung cancer, only patients with alterations to the epidermal growth factor receptor gene respond to tyrosine kinase inhibitors [26]. A drug that targets the cystic fibrosis transmembrane conductance regulator channel activity, ivacaftor, is prescribed for use in  5% of cystic fibrosis patients [27]. Thus technologies such as whole genome or whole exome sequencing (WGS/WES, respectively) to assess genetic variants that can influence treatment efficacy and or impact the likelihood of adverse events have found their utility as genetic testing tools [28, 29]. Genome sequencing in healthy adults for evaluating predisposition can impact primary care. Genome wide association studies (GWAS) of complex diseases have identified associations between sequence variants and a phenotype. However, linking a specific variant to the genes/pathways that determine this association is a bottle neck that impedes the translation of GWAS findings to therapeutic interventions.

Furthermore, determining whether a sequence variant is pathogenic or not depends on determining the functional consequence of the variation. A recent precision medicine prospective cohort study with 1,190 volunteers integrated WGS, comprehensive metabolomics, clinical laboratory tests, family/medical history, and imaging technologies. This study identified at least one genetic variant that is pathogenic or likely pathogenic in 17% of the volunteers, indicating predisposition. Further assessment by a team of clinicians reported genotype to phenotype associations in 11.5% volunteers [30]. Inclusion of a genome scale functional genomics approach such as metabolomics that measures the complete set of metabolites within an organism/cell/tissue with other methods including WGS allowed deep phenotyping to gain useful insights into genetic predisposition in healthy adults.

At some point we need to ask an important question about GWAS; who owns the data? If it is the individual who donated the data, what are the tradeoffs between access to large datasets for research, and potential harm to or violation of the rights and privacy of the donors?

### Algorithms and experimental design

The machine learning algorithm must be matched with the data set being analyzed. Domain experts using an AI and making decisions from the results of the AI analysis must also be well informed of the nature of the AI, its strengths and weaknesses, and how to interpret the results. This is true of statistical and computational analysis tools alike. Each comes with a set of assumptions on the nature of the data for which the analysis process is appropriate and will assure that results are reliable and interpretable. Without these assurances of correct match between the problem and assumptions of the model, the results are meaningless. For example, when using a supervised learning AI, the training data is assumed to accurately depict correct decisions or classifications.

This is where we tread into the potentially dangerous region of algorithms embedding bias into the AI decision process [31] and possibly using the computer to magnify and perpetrate this bias. Prosperi [32] points out the following: “... medical record data are inherently biased, and even the most advanced deep learning’s denoising autoencoders cannot overcome the bias if not handled a priori by design.” In [11], Obermeyer et. al. illustrate how the choice of seemingly effective proxies for ground truth can be the source of bias. Communication between the domain experts and analysis experts is essential here, and both parties need to be present during problem formulation. At least one no-boundary team member who knows how to work across disciplines and detect both human and machine bias might also be needed.

### Interpretation and communication of results

This again requires a no-boundary or convergent team. This crucial step requires that the AI experts and the domain experts communicate with each other. The AI expert again must clearly communicate how the algorithm works and how to interpret the results, and the domain expert must check the results with the accepted foundational theories in the field. This communication is critical for developing trustworthy AI, and the system must include explainability for transparency. They must question the result, especially when inconsistent with existing theory, and try to explain the result using best knowledge of the data provenance, the nature of the algorithm, and the most up to date theories of the domain under investigation. When communicating the results to the domain expert as well as other stakeholders in support of policy or other decisions, the AI bioinformatics team must assure that the communication mode is clear, accurate, and does not perturb important data signals. Including team members with expertise in producing data stories to communicate scientific results should be considered [33].

In precision medicine, the physician or other healthcare worker is often the interface between the artificial intelligence and the patient. It is important to consider the human-computer interaction aspect of this, as well as the interpersonal. The interpretation of results, but particularly the translation to the bedside, calls for nontraditional, no-boundary approaches. Explainable AI, and also incorporating the expertise needed to ensure understanding and compliance from the patient, are essential. Moreover, human expertise and AI are often combined, for example, when a medical doctor uses a cancer therapy recommendation from an AI but also draws on his/her own experience and intuition as an expert. If human intervention is left out, things can go wrong, make no sense, or simply get ridiculous. [1]

To highlight the importance of a diverse no-boundary team of experts, consider that even statistical significance denoted by use of varied *p* value, does not measure the size of an effect or the importance or practical significance of a result. That is, statistical significance is not equivalent to scientific, human, or economic significance. Any effect, no matter how tiny, can produce a small *p* value if the sample size or measurement precision is high enough, and large effects may produce unimpressive *p* values if the sample size is small or measurements are imprecise [34]. The same can be said for significance and accuracy measures for AI analyses.

The components of a precision medicine ecosystem involve the patients, clinicians, electronic health records, clinical laboratories, knowledge resources and tools and researchers [35]. All components of this ecosystem must work together for developing a continuously learning health care, that can integrate knowledge from each patient interaction, for the best patient outcomes. Beyond the existing barriers for integration, one can appreciate the tremendous growth in the availability of different types of patient data, specifically in the domain of genomics, with concomitant complexity of the technologies and analysis methods utilized to generate and interpret this data. There is a need for communication among experts with a variety of expertise in domain specific knowledge for a single unified goal of devising an optimal diagnostic and therapeutic strategies in patient care. This communication is multi-layered with each participant able to carry out discussions with domain-experts as well as non-domain experts.

For example, there is a need to simplify genomics data results for interpretation by clinicians and other health care professionals. A pragmatic solution for this problem could be provided by a combination of natural language processing (NLP) and artificial intelligence, as described recently for exploring, analyzing, and interpreting omics data by next generation analytics. Given the multitude of high throughput technologies that are being utilized in biomedical research, DrBioRight [36], for example, was developed to enable users to perform omics data analysis using a simple online chat interface with single input and output areas. AI is used to translate users intention, identify appropriate data and analysis and select appropriate visualization. Furthermore, DrBioRight collects feedback on its performance for each executed job and uses this information to improve performance of the NLP and AI modules. Thus, next generation analytics that rely on natural language understanding and AI to learn on the job are an attractive option as they can ensure reproducibility, transparency and are envisioned to be compatible with mobile phones and social media, and can support input from all stakeholders from precision medicine ecosystem for open development of tools for patient care.

Cogent interpretation and communication of both processes and results are needed to support clinical practice. Identification of potential impacts, with an assessment of the tradeoffs between potential harm and benefit is needed. For example, data about individuals and groups is important for research, but we must assure that results are robust enough to support sound clinical decisions, and that the patient’s rights of privacy are not violated.

## Open problems

Developing sound ethical AI for precision medicine is a rapidly developing area. Some questions still remain open. In general, more needs to be done to avoid harm to individuals when using electronic analysis. This is especially important in clinical settings. More work needs to be done to support transparency, interpretability, and explainability of algorithms to assure that biomedical users of AI clearly understand the meaning of signals in data that are being reported by AIs that complement traditional statistical biomedical analysis techniques. Mathematicians, statisticians, and data scientists are developing and enhancing modern quasi-experimental [37] and causal inference techniques [38] to provide a means of constructing comparison groups after the data is collected, and to develop theories that tease out the differences between associations and causal relationships in data [38], respectively. There has been a rise in literature regarding the ethics of AI in general [1, 25, 39, 40]. More work is needed to quantify uncertainty of results among algorithms applied to the same data set, to develop precise methods to calculate the likelihood of error in machine learning and deep learning techniques, and develop metrics to determine bias in AI.

Having a gold standard and/or blueprint for the formation of and communication among NBT members working in diverse teams could provide guidance to these teams on how to work effectively. Many experts and scholars are trained ”in the silos” and have not been exposed to strong models for engagement in teams possessing diverse perspectives and expertise. It is clear today that many problems are best solved by employing a balance of human and machine expertise. Better techniques for integrating computational and human expertise is needed to make sense of modern data.

We mentioned earlier in Sect. 2, that inattention to robust and systematic data collection, handling, and interpretation can cause problems. Procedures for maintaining normalized, consistent and correct data need to be improved. We need to better understand the potential of causal AI [41], and determine if this is even possible for existing machine learning approaches [42]. This is an active area of inquiry in statistics that the AI community has also begun to explore. Efforts to strengthen the communication of results, including transparency, explainability, and interpretability, to support the communication of the processes used for analysis are also needed, as are new techniques for visualization and personification of data. More robust techniques for efficiently handling data provenance, and meta data in general, are still needed.

## Conclusion and future directions

This paper highlights a viable solution, based on no-boundary thinking, to address current ethical challenges in integration of AI in precision medicine and to increase the probability of accurate and effective AI results. Our intent is to raise the awareness for increased intentionality in designing ethical AI for precision medicine. There are many challenges that as a research community, we still need to address. For some of these questions, we need to think like philosophers, not like traditional computational scientists, if we are to develop effective and ethically sound solutions. There is a large literature on ethics in AI, algorithmics, and engineering, as well as bioethics. However, most AI researchers and practitioners are completely unaware of it. Consequently, we often start from scratch ignoring tens to thousands of years of prior work, and repeating old, well known mistakes. On the other hand, the philosophers need to think like AI practitioners and users. They often have simplistic views of the technology, and so often address straw man arguments. The boundary between philosophy and AI is an artificial and harmful one and needs to be dissolved. We argue that technologists who develop these algorithms, need to incorporate domain expert’s advice every step of the way from problem formulation to design and model/result interpretation. Utilizing an NBT approach ensures that the diverse perspectives are present at every step to guarantee a more ethical end product.

## Footnotes

1 See the National Science Foundation’s definition of convergence, which is similar, but not yet as thoroughly formulated as no-boundary thinking: https://www.nsf.gov/od/oia/convergence/index.jsp.
